# *Lysyl oxidase-like protein 1* (*LOXL1*) gene polymorphisms and exfoliation glaucoma in a Central European population

**Published:** 2008-05-09

**Authors:** Georg Mossböck, Wilfried Renner, Christoph Faschinger, Otto Schmut, Andreas Wedrich, Martin Weger

**Affiliations:** 1 Department of Ophthalmology, Medical University of Graz, Austria; 2 Clinical Institute of Medical and Chemical Laboratory Diagnostics, Medical University of Graz, Austria

## Abstract

**Purpose:**

Exfoliation syndrome (XFS) is characterized by an accumulation of abnormal extracellular material in the anterior part of the eye that frequently leads to increased intraocular pressure and glaucomatous optic neuropathy. Recently, two non-synonymous polymorphisms (rs1048661 G>T and rs3825942 G>A) of *lysyl oxidase-like protein 1* (*LOXL1*), a monoamine oxidase that catalyzes the polymerization of tropoelastin to elastin, were found to be associated with increased risk for XFS and exfoliation glaucoma (XFG). The aim of the present study was to investigate the role of these *LOXL1* variants in a Central European cohort of Caucasian patients with XFG.

**Methods:**

The present case-control study comprised of 167 unrelated patients with XFG and 170 control subjects. Genotyping of the *LOXL1* rs1048661 and rs3825942 polymorphisms was done using polymerase chain reaction.

**Results:**

The frequency of allele G of rs1048661 as well as rs3825942 was significantly higher in patients than in controls (rs1048661: 0.841 in patients versus 0.669; p<0.001; rs3825942: 0.994 in patients versus 0.817; p<0.001). Odds ratios of 52.1 (95% confidence interval [CI]: 13.85–195.6) and 14.67 (95% CI: 3.81–56.2), respectively, were calculated for the two high-risk haplotypes GG and TG compared to the haplotype GA.

**Conclusions:**

Our data confirm the previously reported association between *LOXL1* polymorphisms and XFG and extend our knowledge to a Central European population.

## Introduction

Exfoliation syndrome (XFS; OMIM 177650) is characterized by an accumulation of abnormal extracellular fibrillar material not only in different structures of the eye but also in various extraocular tissues [1]. Therefore, XFS has been suggested to be a generalized elastic fibrillopathy. Exfoliation fibrils are highly characteristic and consist of a protein core including components of basement membrane and the elastic fiber system surrounded by glycoconjugates [2]. Prevalence of XFS varies widely with the highest prevalence in the Nordic European countries (up to 25%) and 5%–15% in Central European populations [2,3].

Clinically, exfoliation material is recognized as a whitish material on the anterior capsule of the lens and flakes at the pupillary border. Secondary open-angle glaucoma due to XFS (exfoliation glaucoma, XFG) develops as a consequence of deposition of exfoliation material and of liberated iris pigment in the trabecular meshwork leading to elevated intraocular pressure and consecutively glaucomatous optic neuropathy [4].

The exact cause for the production of the exfoliation material is still elusive, but there is ample evidence that genetic factors may contribute to the pathogenesis of XFS. Various family-based studies reported a higher frequency of XFS among relatives of patients with XFS [5-8]. Furthermore, increased rates of loss of heterozygosity have been found in specimens of the anterior segment in individuals with XFS, suggesting genetic factors play a role in the pathogenesis of the disease [9].

Most importantly, a recent genome-wide association study from Icelandic and Swedish patients with XFS and XFG found two common non-synonymous single nucleotide polymorphisms in exon 1 of the *lysyl oxidase-like protein 1* gene (*LOXL1*; OMIM 153456) conferring increased risk for the development of XFS and XFG (rs1048661 and rs3825942) [10]. rs 1048661 induces an amino acid change from arginine to leucine at position 141 (R141L) whereas rs3825942 induces an amino acid change from glycine to asparagine at position 153 (G153D).

So far, the potential role of these polymorphisms has not yet been determined in a Central European population. Therefore, our study was set to investigate the aforementioned *LOXL1* variants in a Central European cohort of Caucasian origin.

## Methods

One hundred sixty-seven unrelated patients with XFG and 170 control subjects were enrolled in the present retrospective case-control study. All study participants were Caucasians from the same geographical area in the southern part of Austria. Participants were seen at the Department of Ophthalmology, Medical University of Graz, Graz, Austria between May 2003 and April 2007 and gave informed, written consent before enrollment. The study was conducted in accordance with the National Gene Technology Act of Austria and the guidelines of the local ethics committee.

Patients with XFG underwent slit lamp biomicroscopy in mydriasis, testing for best corrected visual acuity, Goldmann applanation tonometry, gonioscopy, pachymetry, and standard automated perimetry (Octopus 101, programme G2; Interzeag, Bern, Switzerland) or – in cases of profoundly decreased visual acuity – Goldmann perimetry. Control subjects underwent slit lamp biomicroscopy in mydriasis, Goldmann applanation tonometry, and testing for best corrected visual acuity. Optic discs in all participants were assessed by glaucoma specialists (C.F. and G.M.).

XFG was defined by the presence of typical exfoliation material on the anterior lens capsule, an intraocular pressure of at least 22 mmHg before initiation of a pressure-lowering therapy, an open anterior chamber angle, optic disc changes characteristic for glaucoma (notching, thinning of the neuroretinal rim, and increased cup/disc ratio in relation to the optic disc size), visual field defects characteristic for glaucoma (inferior or superior arcuate scotoma, nasal step, and paracentral scotoma), and the absence of conditions leading to secondary glaucoma.

Control subjects showed biomicroscopically no evidence of exfoliation material on the anterior capsule of the lens and no morphological damage indicative for open-angle or angle closure glaucoma. Control subjects were admitted to our department for cataract surgery.

### Genotype determination

Genomic DNA was extracted from peripheral blood lymphocytes by standard techniques and stored at –70 °C until genotype determination. For genotyping of the *LOXL1* R141L polymorphism, a 179 base-pair segment containing the polymorphic site was amplified by polymerase chain reaction using forward primer 5′-GCC GTC GGG GAC AGC A-3′ and reverse primer 5′-TAG TTC TCG TAC TGG CTG ACG AA-3′. Amplified products were digested with restriction enzyme SmaI (New England Biolabs, Frankfurt, Germany). The 141R allele was cut into two fragments of 151 and 28 base pairs whereas the 141L allele remained uncut. Fragments were separated on 2.5% agarose gels and visualized by use of ethidium bromide.

The *LOXL1* G153D polymorphism was determined by a 5′-exonuclease assay (TaqMan; Applied Biosystems, Vienna, Austria) using forward primer 5′-ACC TCC GTC TCC CAG CAA-3′, reverse primer 5′-TGC TGG CGA AGG CCG AAG-3′, LOXL1–153G-probe VIC-CGG AGC CCC CGT G-NFQ, and LOXL1–153D-probe FAM-AGG CGG AGT CCC CGT-NFQ (Applied Biosystems, Vienna, Austria). The assay was performed according to the manufacturer’s instructions, and end-point fluorescence was measured in a lambda Fluoro 320 plus plate reader (MWG Biotech AG, Ebersberg, Germany) using excitation/emission filters of 485/530 nm and 530/572 nm, respectively. The data were exported into Excel format and depicted and analyzed as a scatter plot.

### Statistical analysis

Descriptive statistics were used to calculate frequencies and percentages of discrete variables. Continuous data are given as mean±standard deviation (SD). Means were compared using the Mann–Whitney test. Proportions of groups were compared by the χ^2^ test. Odds ratio (OR) and 95% confidence interval (95% CI) were calculated by logistic regression. The criterion for statistical significance was p≤0.05. The Hardy–Weinberg (HW) equilibrium has been calculated using HW Diagnostics-Version 1.beta (Fox Chase Cancer Center, Philadelphia, PA). Statistical analysis was done using the SPSS statistical package (SPSS v14.0, Chicago, Illinois) and haplotype analysis was done using Haploview 4.0 [11].

## Results

The present study comprised 167 patients with XFG (91 female, 76 male) and 170 control subjects (95 female, 75 male). The mean age of patients with XFG was 75.7 years (range 50.3–91.6 years), and the mean age of control subjects was 77.1 years (range 58.9–92.7 years). Patients with XFG had a mean deviation of 13.0±6.9 decibel (dB), a mean loss of variance of 36.7±21.5 dB^2^, a mean intraocular pressure of 26.0±11.2 mmHg, and a mean cup disc ratio of 0.80±0.16 in the worse eye.

The success rate of genotyping was 93.5% for rs1048661 and 94.1% for rs3825942. The observed genotype distributions did not deviate from those predicted by the Hardy–Weinberg equilibrium. Table 1 shows the genotype and allele frequencies of rs1048661 and rs3825942 in patients with XFG and control subjects. In both polymorphisms, the prevalence of the GG genotype as well as the frequency of allele G was significantly higher in patients with XFG than in control subjects. An odds ratio of 2.69 (95% CI: 1.59–4.54) was calculated for allele G of rs1048661. For allele G of rs3825942, an odds ratio of 37.29 (95% CI: 6.35–218.02) was calculated.

**Table 1:** Genotype and allele frequencies of rs1048661 and rs3825942 in patients with exfoliation glaucoma and control subjects.

**SNP**	**Patients with XFG (n=167)**	**Control subjects (n=170)**	**p-value**
rs1048661 GG	119 (71.3%)	79 (46.5%)	5.1x10^−6^
GT	43 (25.7%)	70 (41.2%)	
TT	5 (3.0%)	21 (12.4%)	
rs1048661 allele G	0.841	0.671	2.55x10^−7^
rs3825942 GG	165 (98.8%)	109 (64.1%)	9.91x10^−19^
GA	2 (1.2%)	60 (35.3%)	
AA	-	1 (0.6%)	
rs3825942 allele G	0.994	0.817	5.76x10^−15^

Genotype and allele frequencies of rs1048661 and rs3825942 in exon 1 of *LOXL1* in Central European patients with exfoliation glaucoma versus Central European control subjects. Proportions of groups were compared by the χ^2^ test. Numbers for genotypes are n (%).

The two variants are in strong linkage disequilibrium (D’=1.0), and only three possible haplotypes were observed in our cohort (Table 2). Compared to the protective haplotype GA, the GG haplotype conferred an odds ratio of 52.1 (95% CI: 13.85–195.6) while the TG haplotype conferred an odds ratio of 14.67 (95% CI: 3.81–56.2).

**Table 2:** Frequencies of *LOXL1* haplotypes in patients with exfoliation glaucoma and control subjects.

**Haplotype**	**Patients with XFG (n=167)**	**Control subjects (n=170)**	**p-value**
G G	279 (83.5%)	166 (48.8%)	1.86x10^−21^
T G	53 (15.9%)	112 (32.9%)	
G A	2 (0.06%)	62 (18.2%)	

Frequencies of haplotypes of rs1048661 and rs3825942 in exon 1 of *LOXL1* in Central European patients with exfoliation glaucoma versus Central European control subjects. Haplotype analysis was performed using Haploview 4.0. Proportions of groups were compared by the χ^2^ test. Numbers for haplotypes are n (%).

Positive likelihood ratio and negative likelihood ratio were 1.26 and 0.47, respectively, for allele G of rs1048661. For allele G of rs3825942, positive likelihood ratio and negative likelihood ratio were 1.22 and 0.03, respectively.

## Discussion

Mainstay of the pathogenesis of exfoliation syndrome is the accumulation of pathognomonic fibrils in the anterior segment of the eye as well as in extraocular locations [1]. These fibrils are partly composed of components of the elastic fiber system like elastin, tropoelastin, amyloid P, and latent TGF-β binding proteins [2,4]. As a monoamine oxidase secreted by fibrogenic cells, LOXL1 catalyzes the deamination of lysyl residues of tropoelastin, the monomeric form of elastin. This deamination leads to the polymerization of tropoelastin to elastin, which is the first step of elastogenesis [12,13]. Therefore, it is biologically plausible that an altered function of one of the key enzymes of elastogenesis leads to an increased susceptibility for XFS and XFG. Mice lacking LOXL1 display tropoelastin accumulation in multiple tissues, which leads to pelvic organ prolapse, emphysematous changes, and vascular abnormalities [13]. Interestingly, regarding primary open-angle glaucoma, *elastin* and *lysyl oxidase-like protein 2* has been suggested as candidate susceptibility genes [14].

The present study being the first in a Central European population confirms the strong association between *LOXL1* variants and XFG. Beside the original study from Thorleifsson and coworkers [10] that included an Icelandic and a Swedish cohort, four studies from the United States, one study from Australia, one study from Japan, and one from India investigating *LOXL1* polymorphisms in XFS and XFG have been performed [10,15-21]. The prevalence of allele G of rs1048661 and rs3825942 found in the present study were remarkably similar to four of these studies [14-16] while in three studies, slightly lower prevalences have been reported (Table 3) [18-20]. This fact may be due to greater ethnic heterogeneity of the investigated populations or different additional genetic or environmental risk factors. It is noteworthy that in the study from Japan, no allele G in rs1048661 was detected in patients with XFG while frequency of allele G in rs3825942 was found to be 100% in their study [21]. In contrast, in patients with XFG in our cohort frequencies of the G alleles of rs1048661 and rs3825942 was 84.1% and 99.4%, respectively, pointing towards significant differences in the prevalence of rs1048661 among different ethnicities.

**Table 3:** Distribution of frequencies and odds ratios of rs1048661 and rs3825942 among different populations.

**Reference**	**Phenotype**	**Numbers (cases/controls)**	rs1048661 **allele G**	**OR** **(CI 95%)**	rs3825942 **allele G**	**OR** **(CI 95%)**
Thorleifsson et al. [10] Iceland	XFG	75/14474	0.827	2.56 (1.74–3.77)	0.987	13.23 (5.59–31.29)
Thorleifsson et al. [10] Sweden	XFG	198/199	0.834	2.39 (1.72–3.34)	0.995	27.28 (11.44–65.07)
Fingert et al. [15] USA	XFS	72/75	0.819	3.03 (1.78–5.15)	0.986	9.68 (2.44–38.15)
Hayashi et al. [21] Japan	XFG	27/189	0	-	1.0	-
Hewitt et al. [18] Australia	XFG, XFS	86/2087	0.78	1.86 (1.27–2.76)	0.95	3.81 (1.88–9.02)
Yang et al. [16] USA	XFG	49/170	ND		1.0	-
Challa et al. [19] USA	XFG	50/235	0.787	1.86 (1.1–3.15)	0.939	3.05 (1.2–7.76)
Fan et al. [17] USA	XFG	141/80* 146/88 **	0.84	2.06 (1.29–3.3)	0.99	24.77 (7.5–81.83)
Ramprasad et al. [20] India	XFG,XFS	52/97	0.72	1.49 (0.89–2.51)	0.92	4.17 (1.89–9.18)
Mossböck et al. (This report) Central Europe	XFG	167/170	0.841	2.69 (1.59–4.54)	0.994	37.29 (6.35–218.02)

Presented are allele frequencies and odds ratios of the G-allele of rs1048661 and rs3825942 in exon 1 of *LOXL1* in patients with exfoliation syndrome and exfoliation glaucoma among different populations including the present study. The asterisk indicates numbers for rs104866 and the double asterisk indicates numbers for rs3825942; ND=not determined.

With positive and negative likelihood ratios of 1.26 and 0.47 (allele G of rs1048661) and 1.22 and 0.03 (allele G of rs3825942), respectively, genetic testing for XFG based solely on these polymorphisms of *LOXL1* can not be recommended.

In conclusion, the results of our study demonstrate the importance of the *LOXL1* polymorphisms in a Central European population of Caucasian origin. As other genetic or environmental factors are likely to be involved in the pathogenesis of XFG, further studies are warranted.
